# The Common C49620T Polymorphism in the Sulfonylurea Receptor Gene SUR1 (ABCC8) in Patients with Gestational Diabetes and Subsequent Glucose Metabolism Abnormalities

**DOI:** 10.1155/2012/712617

**Published:** 2012-08-15

**Authors:** Piotr Molęda, Agnieszka Bińczak-Kuleta, Katarzyna Homa, Krzysztof Safranow, Zbigniew Celewicz, Anhelli Syrenicz, Adam Stefański, Aneta Fronczyk, Lilianna Majkowska

**Affiliations:** ^1^Department of Diabetology and Internal Diseases, Pomeranian Medical University, Szczecin, Poland; ^2^Chair of Clinical Biochemistry and Laboratory Diagnostics, Pomeranian Medical University, Szczecin, Poland; ^3^Chair of Biochemistry and Medical Chemistry, Pomeranian Medical University, Szczecin, Poland; ^4^Department of Maternal-Fetal Medicine and Gynecology, Pomeranian Medical University, Szczecin, Poland; ^5^Department of Endocrinology, Metabolic and Internal Diseases, Pomeranian Medical University, Szczecin, Poland

## Abstract

*Aim*. The aim of this study is to investigate the relationship between the common C49620T polymorphism in the sulfonylurea receptor (SUR1) gene and glucose metabolism, **β**-cell secretory function and insulin resistance in women with a history of gestational diabetes (GDM). *Material and Methods*. Study group included 199 women, diagnosed GDM within the last 5–12 years and control group of comparable 50 women in whom GDM was excluded during pregnancy. Blood glucose and insulin levels were measured during oral glucose tolerance test. Indices of insulin resistance (HOMA-IR) and **β**-cell function (HOMA %B) were calculated. In all patients, the C49620T polymorphism in intron 15 of the SUR1 gene was determined. *Results*. The distribution of the studied polymorphism in the two groups did not differ from each other (**χ** ^2^ = 0.34, *P* = 0.8425). No association between the distribution of polymorphisms and coexisting glucose metabolism disorders (**χ** ^2^ = 7,13, *P* = 0, 3043) was found. No association was also observed between the polymorphism and HOMA %B or HOMA-IR. *Conclusions*. The polymorphism C49620T in the SUR1 gene is not associated with insulin resistance and/or insulin secretion in women with a history of GDM and does not affect the development of GDM, or the development of glucose intolerance in the studied population.

## 1. Introduction

Gestational diabetes mellitus (GDM) is defined as any degree of glucose intolerance with onset or first recognition during pregnancy [1]. It affects about 7% of Caucasian pregnant women annually and it is the most common metabolic disorder complicating pregnancy [2]. In recent years, an increase in the incidence of GDM has been observed [2, 3]. There is no clear data on the causes of this phenomenon. Perhaps it is due to the higher prevalence of obesity in women of childbearing age, delayed time of the first pregnancy, changing diagnostic criteria for GDM (especially in the USA), and the widespread use of screening tests for GDM. 

A key role in the pathogenesis of GDM is ascribed to insulin resistance increasing during pregnancy, followed by inadequate insulin secretion, in a similar fashion as observed in type 2 diabetes (DM2). After delivery, glucose levels usually return to normal, but GDM diagnosis increases the risk of developing DM2 in the future. It is estimated that 30–70% of women who have a history of GDM will develop DM2 or a prediabetic state within 5–10 years [4, 5]. Key predisposing factors to DM2 in such women include (1) obesity before and after pregnancy, (2) number of pregnancies, (3) age, (4) need for insulin therapy due to GDM, (5) obstetric failure, and (6) a positive family history of DM2 [2, 6–8]. Both insulin resistance and inadequate insulin secretion are considered as possible causes of the increased risk of DM2 [9–12]. It is not clear, however, what is their role in the development of DM2 in women with a history of GDM. 

The vast majority of studies evaluating the incidence and risk of DM2 in women with a history of GDM focused on classic risk factors (anthropometric parameters, family history, and obstetric history). By contrast, the genetic aspects of the development of GDM and subsequent glucose intolerance in women with a history of GDM are not well known.

Genetic factors combined with environmental influences are considered as basic abnormalities in the pathogenesis of DM2. Since a history of GDM is the strongest known risk factor for the development of DM2, many authors believe that GDM and DM2 are closely related disorders or even that the sudden increase in insulin resistance during GDM leads to the occurrence of latent glucose metabolism abnormalities typical for the very early stages of DM2. Therefore, it is possible that similar genetic factors play a role in the pathogenesis of GDM and DM2. Indeed, some of the type 2 diabetes-associated genetic variants discovered in the recent genome-wide association (GWA) studies are also associated with GDM in Korean women [13]. On the other hand, it is also possible that GDM and DM2 are two distinct entities and different genes influence the development of DM2 in women with a history of GDM. For example, associations between SUR1 gene polymorphisms and persistent neonatal diabetes [14], DM2 [15, 16], and GDM [17] have been described, whereas the gene has not been associated with increased DM2 risk in GWA studies performed in the general population. The protein encoded by the SUR1 (ABCC8) gene is a member of the superfamily of ATP-binding cassette transporters and modulates the activity of ATP-sensitive potassium channels and insulin release. Therefore, it might be involved in the impairment of insulin secretion considered as a dominant factor in type 2 diabetes pathogenesis.

In the present study, we assessed relationships between glucose metabolism, *β*-cell secretory function, and insulin resistance in women with a history of GDM in the context of the common C49620T polymorphism in the SUR1 gene.

## 2. Material and Methods

The study group—GDM (+)—was composed of patients attending the outpatient unit of the Department of Diabetology and Internal Diseases at the Pomeranian Medical University (Szczecin, Poland) treated for gestational diabetes between the years 1996 and 2003. About 1200 patients were invited to participate in the study and 204 of them accepted the invitation. Due to the presence of anti-GAD antibodies, 5 women were excluded from further analysis. Eventually, the study group consisted of 199 women, mean age 38.4 ± 6.6 years, who gave birth 5–12 years before the present study and in whom GDM was diagnosed during pregnancy with use of an oral glucose tolerance test (OGTT).

The control group—GDM (−)—consisted of 50 women of comparable age (mean 36.8 ± 5.6 years), who gave birth at the same time, but in whom GDM during pregnancy was excluded. The protocol of the study was accepted by the Bioethical Committee of the Pomeranian Medical University in Szczecin.

After signing a written consent, all participants of the study were interviewed for the number, time and history of their pregnancies. The clinical examination included evaluation of (1) body weight with the calculation of body mass index [BMI (kg/m^2^)], (2) waist and hip circumference with the calculation of waist-hip ratio (WHR), (3) blood pressure (according to the European Society of Hypertension guidelines) [18], (4) heart rate, and (5) fat tissue mass and body fat percentage on the basis of bioelectrical impedance analysis using the Tanita scale SC-330S (Tanita Corporation, Japan).

All patients underwent a 75 g OGTT with measurement of plasma glucose enzymatic method (Cormay SA, Poland), and plasma insulin (IRMA method, BioSource Europe SA, Belgium) at 0, 60 and 120 minutes of the test. The homeostasis model assessment (HOMA) was used to evaluate insulin resistance (HOMA-IR), insulin sensitivity indexes (HOMA %S) and steady state beta-cell function—(HOMA %B) (the HOMA Calculator—software v2.2.2) [19].

Glycated haemoglobin—HbA1c (HPLC method, BIO-RAD Laboratories, Germany)—was measured and titers of antibodies against glutamic acid decarboxylase (anti-GAD ELISA method, Euroimmun, Germany) were assessed in all participants of the study to exclude type 1 diabetes cases.

In addition, in all patients, the C49620T polymorphism in intron 15 of the SUR1 (ABCC8) gene was determined.

### 2.1. Genotyping

Genotyping was performed using a method described previously [20]. Genomic DNA was isolated from peripheral blood leukocytes using a nonenzymatic and non-organic method with the use of a 2% Triton X-100 solution. The following pair of primers was used for DNA amplification by PCR: 5′-TTGGGTGCATCTGTCTGTCTGTCTTT-3′ as the sense primer and 5′-AGCCCACCTGCCCCACGAT-3′ as the antisense primer (GenBank accession L78222). These primers yielded PCR product of 122 bp in length. Subsequently, the amplicons were digested with the restriction enzyme *Pst I* and separated by 3% agarose gel electrophoresis stained with ethidium bromide. The *Pst I* cleaved the wild-type allele (C49620), generating 2 fragments (88 and 34 in length, resp.), but did not cut the T49620 allele. The results were recorded with photographs of gels in UV light and all samples were independently genotyped on a blind basis in duplicate.

### 2.2. Statistical Analysis

The software STATISTICA version 7.1 (StatSoft Inc., Tulsa, OK, USA) was used for database management and statistical analysis. The Mann-Whitney test and *χ* ^2^-test were used for comparison of continuous and nominal variables, respectively. Correlations between continuous variables in each group were analyzed using Spearman's rank correlation coefficient (Rs). ABCC8 genotype and allele frequencies as well as compliance of their distribution with the Hardy-Weinberg principle were analyzed by the *χ* ^2^ test or Fisher's exact test. To evaluate the association of genotype with the presence of GDM, odds ratio (OR) and 95% confidence interval (95% CI) were calculated. The *P* value of <0.05 was considered to be statistically significant. 

## 3. Results

The study group did not differ from the control groups in terms of age and anthropometric parameters (body weight, BMI, waist and hip circumferences, WHR, fat tissue mass, and lean body mass). There was also no difference in systolic, diastolic blood pressure, and pulse rate between the groups (Table 1). Results of the OGTT are presented in Table 1. In women with a history of GDM, fasting glucose levels at the 60th and 120th minute of the test were significantly higher than in control individuals. In addition, in the study group, higher plasma insulin levels during the OGTT were observed, whereas they were similar in basal conditions. The study group also was characterized by significantly higher HbA1c values. With regard to insulin sensitivity, HOMA-IR and HOMA %S parameters were similar in both groups, whereas HOMA %B was found to be significantly lower in the study group.

**Figure 1:**
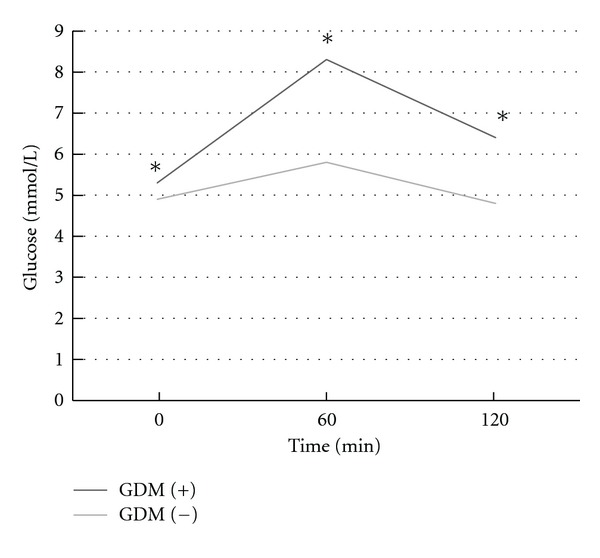
The oral glucose tolerance test results in the study group (*P < 0.001).

Based on the results of OGTT, patients were classified into subgroups with (1) normal glucose tolerance (NGT), (2) impaired fasting glucose (IFG), (3) impaired glucose tolerance (IGT), and (4) diabetes (DM2) defined according to WHO guidelines [1]. In the GDM (+) group NGT was observed in 113 (56.8%), IFG in 40 (20.1%), IGT in 33 (16.6%), and DM2 in 13 (6.5%) patients. In the GDM (−) group NGT was observed in 44 (88.0%), IFG in 5 (10.0%), and IGT in 1 (2.0%) subjects. None of persons from the GDM (−) group fulfilled diabetes criteria. The frequency of the occurrence of the above mentioned carbohydrate metabolism disorders was significantly different between the two study groups (*χ* ^2^ = 18.7, *P* = 0.0003). The results are shown in Table 2 and Figure 1.

All results were compared between subgroups of patients, classified either according to genotype (CC homozygotes versus CT heterozygotes versus TT homozygotes) or to variant carriage: C allele carriers versus subjects without C allele (CC + CT versus TT), and T allele carriers (CT + TT) versus wild-type CC homozygotes. The distribution of the C49620T polymorphism of the SUR1 gene in both investigated groups is shown in Table 3. The distribution of the polymorphism in the two groups did not diverge significantly from the Hardy-Weinberg equilibrium (*P* = 0.5773 for the study group and *P* = 0.4046 for the control group) and did not differ from each other (*χ* ^2^ = 0.34, *P* = 0.84252). 

No association was found between the studied polymorphism and anthropometric parameters for both analyzed groups. In patients with a history of GDM, plasma glucose and insulin levels were higher in genotype CT and TT carriers compared to genotype CC carriers, mostly on the border of statistical significance. Only in the case of insulin at 120 min the difference was significant (CT versus CC, *P* < 0.05). No association was also observed between the polymorphism and parameters describing beta-cell function (HOMA %B) or insulin sensitivity (HOMA-IR, HOMA %S) (Table 4).

The relationship between the studied polymorphism and OGTT results was assessed. We found no association between the distribution of polymorphisms and coexisting glucose metabolism disorders (*χ* ^2^ = 7.13, *P* = 0.30435) (Table 5).

## 4. Discussion

The main finding of the present study was significantly impaired pancreatic *β*-cell function in women with a history of GDM, while their insulin resistance index (HOMA-IR) was not different from that of the control group. It suggests that it is abnormalities of insulin secretion rather than changes in insulin resistance that is crucial for the development of DM2 in this population.

It is assumed that the natural history of diabetes leads from the initially dominant insulin resistance and compensatory hyperinsulinemia to the exhaustion of *β*-cells and overt hyperglycemia. The involvement of these components is very individual, but recently more attention is directed towards the early impairment of insulin secretion as a dominant factor in DM2 pathogenesis [11, 12].

A history of GDM is an important risk factor for DM2. Many authors believe that GDM and DM2 are closely related disorders. The risk of developing DM2 in the population of women with a history of GDM is estimated to be 30–50%, or even 70% [4, 21]. Therefore, it is the strongest known risk factor.

In our work, we tried to determine which factor is more important in the development of DM2 in women with a history of GDM—insulin resistance or defective insulin secretion. In addition, we evaluated the relationship between different parameters of glucose metabolism and the C49620T polymorphism in the SUR1 (ABCC8) gene, of which the relationship with DM2 had been previously described [22, 23].

The study and control groups did not differ in terms of age, body weight, waist circumference, and the fat tissue mass and content. The ratio of individuals with normal and increased body weight was also similar in both groups. However, glucose metabolism abnormalities were significantly more common in the GDM (+) group. Since the insulin resistance index (HOMA-IR) did not differ between the groups, it seems that insulin resistance in peripheral tissues is not a dominant factor leading to glucose metabolism disorders in this population. In contrast, significantly impaired pancreatic *β*-cell function evaluated with use of the HOMA %B index suggests that abnormalities of insulin secretion in women with a history of GDM may be crucial in the pathogenesis of DM2. There exist studies linking a family history of GDM with subsequent onset of type 1 or type 2 diabetes [24, 25]. The determination of the genetic background of GDM may be essential to answer the question whether it is a distinct clinical entity or merely an early phase of other types of diabetes. Genetically determined diabetes has relatively low prevalence and its diagnostics is not easy in practice, and also not very common. Therefore, it is difficult to identify families with an increased risk of that form of the disease. Other problems arise from various diagnostic criteria of the condition, which have been changed over time, and heterogeneous guidelines for GDM screening in different countries. Additional difficulties are related to environmental influences and racial differences [26].

There are many genes candidates for GDM pathogenesis, including those associated with insulin secretion, insulin resistance, and obesity. For example, a high incidence of various mutations in the glucokinase gene has been shown in patients with GDM [27, 28]. Watanabe et al. demonstrated significantly more frequent occurrence of mutations in the hepatocyte nuclear factor-4*α* gene (HNF4A) P2 promoter in GDM women of Mexican origin [29]. Both mentioned genes are associated with monogenic forms of diabetes. Ober et al. found more frequent mutations in the insulin receptor gene in patients with a history of GDM. Recently, Lauenborg et al. analyzed 11 genes associated with an increased risk of type 2 diabetes (TCF7L2, CDKAL1, SLC30A8, HHEX-IDE CDKN2A/2B, IGF2BP2, FTO, TCF2, PPAR*γ*, WFS1, and KCNJ11) in the group of 283 women with a history of GDM. All tested variants were significantly more frequent in the studied population, which might confirm a common genetic background of GDM and DM2 [30].

There are not many works studying the relationship between ABCC8 gene polymorphisms and GDM. Rissanen et al. investigated possible associations of the variants in the nucleotide-binding fold regions of the ABCC8 gene with GDM and DM2 in Finnish subjects. They found that the atagGCC allele of exon 16 splice acceptor site and an AGG allele of the R1273R polymorphism were more frequent in subjects with GDM and DM2 than in normoglycemic subjects. However, the variants of the ABCC8 gene were not associated with altered first-phase insulin secretion in normoglycemic subjects (18). In a small study, Niu and colleagues also demonstrated an association between −3t→c and A/G polymorphisms and incidence of GDM and DM2 in the Chinese population [31]. In turn, Ackermann et al. found that apoptosis induced by 17*β*-estradiol in islets and cells expressing different forms of the sulfonylurea receptor can be influenced by certain SUR1 mutations (M1289T, R1379C, and R1379L). This phenomenon would explain the abnormal secretion of insulin during pregnancy in carriers of these polymorphisms [32]. 

Our results, however, do not confirm these earlier observations. It seems that the C49620T polymorphism of the ABCC8 gene is not related to GDM and to impaired insulin secretion observed in women with a history of GDM.

## 5. Conclusions

1. A significant defect of insulin secretion occurs in women with a history of gestational diabetes, whereas insulin sensitivity remains unchanged.
2. The polymorphism C49620T in the SUR1 (ABCC8) gene is not associated with insulin resistance and/or insulin secretion in women with a history of GDM.
3. It seems that the polymorphism C49620T in SUR1 (ABCC8) gene does not affect the development of GDM, or the development of glucose intolerance in the studied population.

## Figures and Tables

**Table 1:** Clinical characteristics of the study groups and glucose metabolism, beta-cell function, and insulin resistance in baseline conditions and during the oral glucose tolerance test.

	GDM (+)	GDM (−)	
Parameter	Mean ± SD	Mean ± SD	*P* value
	(*n* = 199)	(*n* = 50)	
Number of pregnancies	2.2 ± 2.8	1.9 ± 2.1	NS
Number of deliveries	1.88 ± 2.8	1.82 ± 2.4	NS
Average time since the GDM-complicated pregnancy (years)	7.4 ± 0.7	7.8 ± 1.0	NS
Age at the time of delivery (years)	30.8 ± 5.7	29.2 ± 6.1	NS
Age (years)	38.4 ± 6.6	36.8 ± 5.6	NS
Height (cm)	163.3 ± 5.9	164.2 ± 6.8	NS
Body weight (kg)	67.9 ± 15.2	68.7 ± 14.9	NS
BMI (kg/m^2^)	25.5 ± 5.6	25.4 ± 5.0	NS
Waist circumference (cm)	84.8 ± 13.2	85.0 ± 13.4	NS
Hip circumference (cm)	98.5 ± 11.1	100.1 ± 10.7	NS
WHR	0.86 ± 0.09	0.84 ± 0.07	NS
Fat tissue mass (kg)	21.9 ± 10.2	22.6 ± 11.1	NS
% of fat tissue (%)	31.0 ± 8.0	31.2 ± 8.9	NS
Lean body mass (kg)	45.5 ± 6.1	46.1 ± 4.8	NS
Mean systolic blood pressure (mm Hg)	123.2 ± 17.3	118.8 ± 14.8	NS
Mean diastolic blood pressure (mm Hg)	81.4 ± 12.0	79.0 ± 9.9	NS
Heart rate (/min.)	78.9 ± 11.5	80.2 ± 10.8	NS
Glucose 0^′^ (mmol/L)	5.3 ± 0.7	4.9 ± 0.6	<0.0001
Glucose 60^′^ (mmol/L)	8.3 ± 2.6	5.8 ± 1.9	<0.0001
Glucose 120^′^ (mmol/L)	6.4 ± 2.2	4.8 ± 1.1	<0.0001
Insulin 0^′^ (*μ*IU/mL)	13.7 ± 8.7	13.7 ± 8.5	NS
Insulin 60^′^ (*μ*IU/mL)	106.8 ± 62.6	83.8 ± 41.1	0.0204
Insulin 120^′^ (*μ*IU/mL)	74.6 ± 58.7	47.5 ± 30.6	0.0013
HbA1c (%)	5.6 ± 0.4	5.4 ± 0.4	0.0083
HOMA IR	1.76 ± 1.05	1.73 ± 1.03	NS
HOMA %S	72.2 ± 38.5	69.7 ± 27.0	NS
HOMA %B	125.4 ± 52.7	145.7 ± 49.2	0.0012

**Table 2:** The oral glucose tolerance test results.

	GDM (+)	GDM (−)
	Mean ± SD (*n* = 199)	Mean ± SD (*n* = 50)
NGT	113 (56.8%)	44 (88.0%)
IFG	40 (20.1%)	5 (10.0%)
IGT	33 (16.6%)	1 (2.0%)
DM2	13 (6.5%)	0 (0%)

**Table 3:** Polymorphism C49620T in the SUR1 (ABCC8) gene distribution.

	GDM (+) *n* = 199 (%)	GDM (−) *n* = 50 (%)	*P* value
CC	54 (27.14%)	14 (28.0%)	NS
CT	96 (48.24%)	22 (44.0%)	NS
TT	49 (24.62%)	14 (28.0%)	NS
C allel carriers	204 (51.3%)	50 (50.0%)	NS
T allel carriers	194 (48.7%)	50 (50.0%)	NS

**Table 4:** Relationships between the polymorphism C49620T in the SUR1 (ABCC8) gene and glucose metabolism parameters, *β*-cell function, and insulin resistance in the GDM (+) group.

Parameter	CC Mean ± SD (*n* = 54)	CT Mean ± SD (*n* = 96)	TT Mean ± SD (*n* = 49)	*P* value CC versus CT	*P* value CT versus TT	*P* value CC versus TT
Glucose 0^′^ (mmol/L)	5.3 ± 0.8	5.3 ± 0.7	5.4 ± 0.7	NS	NS	NS
Glucose 60^′^ (mmol/L)	7.9 ± 2.3	8.5 ± 2.7	8.4 ± 2.5	0.0121	NS	NS
Glucose 120^′^ (mmol/L)	6.2 ± 2.1	6.6 ± 2.3	6.2 ± 2.1	NS	NS	NS
Insulin 0^′^ (*μ*IU/mL)	12.5 ± 6.1	13.6 ± 8.5	15.0 ± 11.2	NS	NS	NS
Insulin 60^′^ (*μ*IU/mL)	96.7 ± 43.9	109.9 ± 64.1	112.0 ± 76.4	NS	NS	NS
Insulin 120^′^ (*μ*IU/mL)	64.3 ± 48.6	78.4 ± 54.4	78.5 ± 75.4	0.0483	NS	NS
HbA1c (%)	5.6 ± 0.5	5.7 ± 0.4	5.7 ± 0.4	NS	NS	NS
HOMA IR	1.63 ± 0.80	1.77 ± 1.08	1.91 ± 1.22	NS	NS	NS
HOMA %B	121.9 ± 48.0	126.3 ± 51.9	126.8 ± 60.1	NS	NS	NS
HOMA %S	77.4 ± 46.3	71.2 ± 31.6	69.1 ± 41.8	NS	NS	NS

**Table 5:** Relationship between the studied polymorphism and OGTT results in the GDM (+) group.

	CC	CT	TT
	*n* = 54 (%)	*n* = 96 (%)	*n* = 49 (%)
NGT	31 (57.4%)	57 (59.4%)	25 (51.0%)
IFT	12 (22.2%)	14 (14.6%)	14 (28.6%)
IGT	9 (16.7%)	15 (15.6%)	9 (18.4%)
DM2	2 (3.7%)	10 (10.4%)	1 (2.0%)

## Abbreviations

WHR: waist/hip ratio
HbA1c: glycated haemoglobin A1c
HOMA-IR: the homeostasis model assessment insulin resistance index
HOMA %S: the homeostasis model assessment insulin sensitivity index
HOMA %B: the homeostasis model assessment beta-cell function
GDM: gestational diabetes mellitus
NGT: normal glucose tolerance
IFG: impaired fasting glucose
IGT: impaired glucose tolerance
DM2: type 2 diabetes mellitus
SUR 1: sulfonylurea receptor type 1.

## References

[1] World Health Organization (1999). *Definition, Diagnosis and Classification of Diabetes Mellitus and its Complications: Report of a WHO Consultation. Part 1: Diagnosis and Classification of Diabetes Mellitus*.

[2] Buchanan TA, Xiang AH (2005). Gestational diabetes mellitus. *Journal of Clinical Investigation*.

[3] Ferrara A (2007). Increasing prevalence of gestational diabetes mellitus: a public health perspective. *Diabetes Care*.

[4] Feig DS, Zinman B, Wang X, Hux JE (2008). Risk of development of diabetes mellitus after diagnosis of gestational diabetes. *Canadian Medical Association Journal*.

[5] Zonenberg A, Telejko B, Topolska J (2006). Factors predisposing to disturbed carbohydrate tolerance in patients with previous gestational diabetes mellitus. *Diabetologia Doswiadczalna i Kliniczna*.

[6] Baptiste-Roberts K, Barone BB, Gary TL (2009). Risk factors for type 2 diabetes among women with gestational diabetes: a systematic review. *American Journal of Medicine*.

[7] Bellamy L, Casas JP, Hingorani AD, Williams D (2009). Type 2 diabetes mellitus after gestational diabetes: a systematic review and meta-analysis. *The Lancet*.

[8] Lauenborg J, Hansen T, Jensen DM (2004). Increasing incidence of diabetes after gestational diabetes. *Diabetes Care*.

[9] Buchanan TA, Metzger BE, Freinkel N, Bergman RN (1990). Insulin sensitivity and B-cell responsiveness to glucose during late pregnancy in lean and moderately obese women with normal glucose tolerance or mild gestational diabetes. *American Journal of Obstetrics and Gynecology*.

[10] Buchanan TA (2001). Pancreatic B-cell defects in gestational diabetes: Implications for the pathogenesis and prevention of type 2 diabetes. *Journal of Clinical Endocrinology and Metabolism*.

[11] Pimenta WP, Calderon IMP, Cruz NS, Santos ML, Aragon FF, Padovani CR (2004). Subclinical abnormalities of glucose metabolism in Brazilian women with a history of gestational diabetes mellitus. *Acta Obstetricia et Gynecologica Scandinavica*.

[12] Kousta E, Lawrence NJ, Godsland IF (2007). Early metabolic defects following gestational diabetes in three ethnic groups of anti-GAD antibodies negative women with normal fasting glucose. *Hormones*.

[13] Cho YM, Kim TH, Lim S (2009). Type 2 diabetes-associated genetic variants discovered in the recent genome-wide association studies are related to gestational diabetes mellitus in the Korean population. *Diabetologia*.

[14] Thomas PM, Cote GJ, Wohllk N (1995). Mutations in the sulfonylurea receptor gene in familial persistent hyperinsulinemic hypoglycemia of infancy. *Science*.

[15] Hani EH, Clément K, Velho G (1997). Genetic studies of the sulfonylurea receptor gene locus in NIDDM and in morbid obesity among French Caucasians. *Diabetes*.

[16] Hart LM, De Knijff P, Dekker JM (1999). Variants in the sulphonylurea receptor gene: Association of the exon 16- 3t variant with Type II diabetes mellitus in Dutch Caucasians. *Diabetologia*.

[17] Rissanen J, Markkanen A, Kärkkäinen P (2000). Sulfonylurea receptor I gene variants are associated with gestational diabetes and type 2 diabetes but not with altered secretion of insulin. *Diabetes Care*.

[18] Mancia G, De Backer G, Dominiczak A (2007). 2007 Guidelines for the Management of Arterial Hypertension: The Task Force for the Management of Arterial Hypertension of the European Society of Hypertension (ESH) and of the European Society of Cardiology (ESC). *Journal of Hypertension*.

[19] http://www.dtu.ox.ac.uk/index.php?maindoc=/homa/index.php.

[20] Stefanski A, Majkowska L, Ciechanowicz A (2007). The common C49620T polymorphism in the sulfonylurea receptor gene (ABCC8), pancreatic beta cell function and long-term diabetic complications in obese patients with long-lasting type 2 diabetes mellitus. *Experimental and Clinical Endocrinology and Diabetes*.

[21] Kim C, Newton KM, Knopp RH (2002). Gestational diabetes and the incidence of type 2 diabetes: a systematic review. *Diabetes Care*.

[22] Inoue H, Ferrer J, Welling CM (1996). Sequence variants in the sulfonylurea receptor (SUR) gene are associated with NIDDM in Caucasians. *Diabetes*.

[23] Laukkanen O, Pihlajamäki J, Lindström J (2004). Polymorphisms of the SUR1 (ABCC8) and Kir6.2 (KCNJ11) genes predict the conversion from impaired glucose tolerance to type 2 diabetes. The Finnish Diabetes Prevention study. *Journal of Clinical Endocrinology and Metabolism*.

[24] Watanabe RM, Black MH, Xiang AH, Allayee H, Lawrence JM, Buchanan TA (2007). Genetics of gestational diabetes mellitus and type 2 diabetes. *Diabetes Care*.

[25] Dorner G, Plagemann A, Reinagel H (1987). Familial diabetes aggregation in type I diabetics: Gestational diabetes an apparent risk factor for increased diabetes susceptibility in the offspring. *Experimental and Clinical Endocrinology*.

[26] McLellan JAS, Barrow BA, Levy JC (1995). Prevalence of diabetes mellitus and impaired glucose tolerance in parents of women with gestational diabetes. *Diabetologia*.

[27] Saker PJ, Hattersley AT, Barrow B (1996). High prevalence of a missense mutation of the glucokinase gene in gestational diabetic patients due to a founder-effect in a local population. *Diabetologia*.

[28] Ellard S, Beards F, Allen LIS (2000). A high prevalence of glucokinase mutations in gestational diabetic subjects selected by clinical criteria. *Diabetologia*.

[29] Watanabe RM, Xiang AH, Allayee H (2003). Evidence of genetic predisposition for B-cell dysfunction in Mexican-American families of probands with gestational diabetes (Abstract). *Diabetes*.

[30] Lauenborg J, Grarup N, Damm P (2009). Common type 2 diabetes risk gene variants associate with gestational diabetes. *Journal of Clinical Endocrinology and Metabolism*.

[31] Niu XM, Yang H, Zhang HY (2005). Study on association between gestational diabetes mellitus and sulfonylurea receptor-1 gene polymorphism. *Zhonghua Fu Chan Ke Za Zhi*.

[32] Ackermann S, Hiller S, Osswald H, Lösle M, Grenz A, Hambrock A (2009). 17*β*-estradiol modulates apoptosis in pancreatic *β*-cells by specific involvement of the sulfonylurea receptor (SUR) isoform SUR1. *Journal of Biological Chemistry*.

